# Exploring Voluntary Vaccinating Behaviors using Evolutionary N-person Threshold Games

**DOI:** 10.1038/s41598-017-16680-z

**Published:** 2017-11-27

**Authors:** Benyun Shi, Weihao Wang, Hongjun Qiu, Yu-Wang Chen, Shaoliang Peng

**Affiliations:** 1 School of Cyberspace, Hangzhou Dianzi University, Hangzhou, 310018 China; 2 School of Information Engineering, Nanjing University of Finance & Economics, Nanjing, 210003 China; 3 Decision and Cognitive Sciences Research Centre, The University of Manchester, Manchester, M13 9SS UK; 4 School of Computer Science, National University of Defense Technology, Changsha, 410073 China; 5 College of Computer Science and Electronic Engineering & National Supercomputer Centre in Changsha, Hunan University, Changsha, 410082 China

## Abstract

Understanding individuals’ voluntary vaccinating behaviors plays essential roles in making vaccination policies for many vaccinepreventable diseases. Usually, individuals decide whether to vaccinate through evaluating the relative cost of vaccination and infection according to their own interests. Mounting evidence shows that the best vaccine coverage level for the population as a whole can hardly be achieved due to the effects of herd immunity. In this paper, taking into consideration the herd immunity threshold, we present an evolutionary N-person threshold game, where individuals can dynamically adjust their vaccinating strategies and their payoffs depend nonlinearly on whether or not the herd immunity threshold is reached. First, in well-mixed populations, we analyze the relationships at equilibrium among the fraction of vaccinated individuals, the population size, the basic reproduction number and the relative cost of vaccination and infection. Then, we carry out simulations on four types of complex networks to explore the evolutionary dynamics of the N-person threshold game in structured populations. Specifically, we investigate the effects of disease severity and population structure on the vaccine coverage for different relative costs of vaccination and infection. The results and findings can offer new insight into designing incentive-based vaccination policies for disease intervention and control.

## Introduction

In epidemiology, extensive efforts have been taken to determine what proportion of individuals need to be vaccinated to prevent epidemics of vaccine-preventable diseases^1–5^. To achieve the critical vaccine coverage, various vaccination policies have been suggested, ranging from preemptive mass vaccination^6^, post-outbreak ring vaccination^7,8^, to mandatory vaccination^9^, among which voluntary vaccination takes into consideration individual strategic behaviors in response to disease epidemic^10–12^. However, due to the effects of herd immunity^13–16^, voluntary vaccination is faced with a long-standing dilemma: the vaccine coverage level achieved through self-interested individuals may differ from what is best for the population as a whole^17^. Therefore, in addition to determine the necessary vaccine coverage for disease eradication, it is also essential to explore individuals’ voluntary vaccinating behaviors under different circumstances so as to help public health authorities design effective incentive-based vaccination polices.

In recent years, the game-theoretic approach has been extensively adopted to study the above-mentioned vaccination dilemma in well-mixed populations^17–23^, where any two individuals meet equally often with each other. For example, Bauch *et al*. have proposed a vaccination game to model the interplay between human vaccinating behaviors and the epidemiological characteristics of the disease^18^. They have revealed that there is a clash between individuals’ self-interest and group interest with respect to smallpox vaccination, which makes voluntary vaccination fail to eradicate a vaccine-preventable disease unless a risk-free vaccination is used^17^. Moreover, they have pointed out that the oscillation of vaccine update is more likely in populations where individuals imitate others more readily or exhibit a strong response to disease prevalence^19,20^. Inspired by minority game methodology, Vardavas *et al*. have found that flu-like seasonal epidemic is unlikely to be prevented through voluntary vaccination even with risk-free vaccine^22^.

While in structured populations, the effectiveness of voluntary vaccination is very different. For example, Perisic and Bauch have shown that disease eradicability through voluntary vaccination depends partially on whether the disease is transmissible only to a few close social contacts of the population^24^. They have demonstrated that social contact structure can change individuals’ incentive to vaccinate and enable disease eradication^25^. Along this line, many studies have focused on investigating the effects of population structure (i.e., social diversity) on human voluntary vaccinating behaviors^26–33^. A variety of network-based frameworks have been proposed to simulate the interplay between disease epidemic and human behavioral responses^34–39^.

Under voluntary vaccination, individuals are assumed to act according to pure self-interest, where they attempt to maximize their own payoffs through weighing the cost of infection^40,41^, over the cost of vaccination (e.g., economic loss^42–44^, and side effects^45–48^). Moreover, in a population, the vaccinating decision of an individual can dynamically evolve, and depend on the decisions made by the rest of the population. In this case, if we treat individuals who take vaccine as *cooperators*, and those who refuse to vaccinate as *defectors*, we can then study the emergence and evolution of individuals’ cooperative behaviors from the perspective of evolutionary game theory. In epidemiology, because whether or not the critical vaccine coverage can be reached is determined through individuals’ collective behaviors, the framework of evolutionary N-person games becomes a natural choice. Taking into consideration of the herd immunity threshold (*HIT*), in this paper, we present an evolutionary N-person threshold game to investigate human voluntary vaccinating behaviors in the face of disease epidemic, where an individual’s vaccinating decision depends not only on the relative cost of vaccination and infection, but also the reachability of the *HIT* in the population.

In many real-world situations, it is often the case that no common benefit can be produced unless a minimum number of *M* individuals decides to cooperate. For example, in an N-person snowdrift game^49,50^, if individuals do not have the ability to clear the snow alone, at least *M* ≥ 2 individuals are required to cooperate with each other to shovel the snow. In this case, the more individuals cooperate, the less effort each one needs to contribute. While in this paper, the proposed N-person threshold game under voluntary vaccination is totally different: The major difference lies in that under the N-person snowdrift games, cooperators will collaboratively share the required cost *c*; in other words, the cost for each cooperator is *c*/*k* if there are *M* ≤ *k* ≤ *N* cooperators. While for the threshold game in this paper, the cost of cooperation (i.e., vaccination) for each individual is always *c* irrespective of the number of cooperators. The second difference is the definition of threshold. In previous studies, the threshold *M* can be arbitrarily defined to evaluate the performance of an evolutionary game. While in this paper, the herd immunity threshold is closely related to the population size and the basic reproduction number *R*_0_ of the disease.

In this paper, we captures the evolution of individuals’ voluntary vaccinating behaviors in the following way. First, we conduct equilibrium analysis on the evolutionary N-person threshold game in well-mixed populations. Theoretically, we reveal the relationships among the fraction of vaccinated individuals at equilibrium, the population size, the relative cost of vaccination and infection, and the reproduction number *R*_0_ of the disease. Then, we carry out simulations on four types of complex networks to investigate the evolutionary dynamics of the N-person threshold game in structured populations. Since people are often structured in groups (e.g., families and colleagues), we assume that each individual together with his/her neighbors in a network forms a locally-mixed group^33,34,51^. Individuals within a group are homogeneously mixing and adjust their vaccinating decisions through imitating one of his/her neighbor’s strategies in an evolutionary process^52–54^. In doing so, the proposed model and method in this paper can not only offer a new perspective for exploring the evolutionary dynamics of human voluntary vaccinating behaviors, but also provide a new type of threshold games to investigate the emergence and evolution of cooperative behaviors in human society.

## Results

We first demonstrate the analytical results about the fraction of vaccinated individuals at equilibrium under the proposed N-person threshold game in well-mixed populations. The effects of the population size and the basic reproduction number *R*_0_ on the stable equilibrium are analyzed with respect to different the cost of vaccination and infection *c* = *c*_*v*_/*c*_*i*_. Then, we carry out simulations on four types of complex networks, which are regular networks, random regular networks, small-world networks, and scale-free networks, to explore the evolutionary dynamics of human vaccinating behaviors in structured populations. Specifically, we investigate the effects of disease severity (i.e., the basic reproduction number *R*_0_) and population structure (i.e., network structure and average degree) on the final vaccine coverage level under different settings of the proposed N-person threshold game. All simulations results are averaged over 50 independent runs for each type of network.

### Analytical results in well-mixed populations

**Figure 1:**
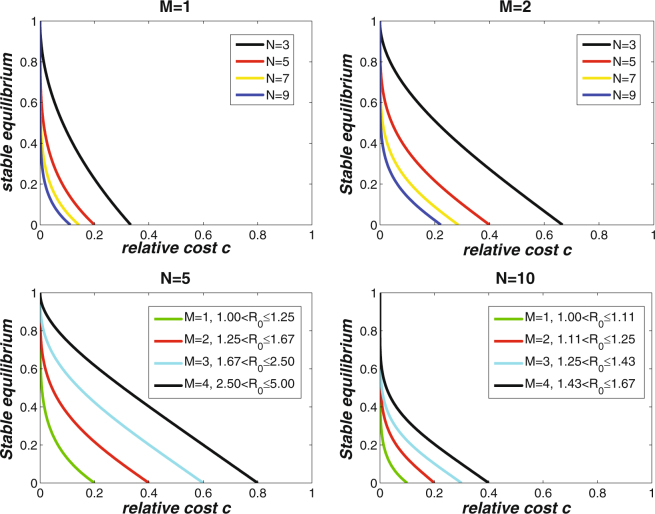
The effects of herd immunity threshold and population size on the fraction of vaccinated individuals at equilibrium. The upper two figures show the effects of population size under the threshold *M* = 1 and *M* = 2, respectively. The following two figures show the effects of threshold and basic reproduction number *R*_0_ when the population size *N* = 5 and *N* = 10, respectively. It can be observed that as the relative cost *c* increases, the fraction of vaccinated individuals at equilibrium decreases nonlinearly for fixed population size *N* and threshold *M*. When the relative cost *c* is large enough, unvaccinated individuals will dominate the whole population.

We obtain the vaccine coverage level (i.e., the fraction of vaccinated individuals) at equilibrium with respect to different relative costs *c* (i.e., *c* = *c*_*v*_/*c*_*i*_) in well-mixed populations. Based on the definition of the herd immunity threshold (see the Method section for details), the critical threshold *M* in our proposed game has a strong relationship with the population size *N* and the basic reproduction number *R*_0_, that is $M=\lceil N\cdot {p}_{c}\rceil =\lceil N\mathrm{(1}-\mathrm{1/}{R}_{0})\rceil$. Given the population size *N* and the basic reproduction number *R*_0_, the threshold *M* can be uniquely determined. Figure 1 shows the effects of *HIT* and population size on the vaccine coverage level at equilibrium, where all the curves are generated based on Equation (9) in Method section. It can be observed that as the relative cost *c* increases, the vaccine coverage at equilibrium decreases nonlinearly for fixed population size *N* and threshold *M*. When the relative cost *c* is large enough, unvaccinated individuals will dominate the whole population. This phenomenon is consistent with the real-world situation: when the infection risk of a disease is low but the side effect of the vaccine is high, most people will not choose vaccination.

Specifically, it can also be observed that for a fixed threshold *M*, the vaccine coverage level at equilibrium decreases as the population size *N* increases (see the upper two figures in Fig. 1). Moreover, when the threshold *M* gets larger, the critical value of relative cost *c* increases to maintain certain vaccine coverage level. With respect to the varying threshold *M*, it can be observed that from the bottom two figures that, the vaccine coverage level at equilibrium increases as the threshold *M* increases given a population size *N*. Each threshold value corresponds to a range of basic reproduction number, which defines the severity of an epidemic. For example, when the population size *N* = 5, the threshold *M* = 4 corresponds to the basic reproduction number $R\in \mathrm{(2.5,5.0])}$. In this case, when an epidemic is much more serious, the vaccine coverage level will become larger through voluntary vaccination. However, the larger the population size, the lower the vaccine coverage level at equilibrium. This finding is consistent with the observation that when neighborhood size is small, rational vaccinating behavior results in rapid containment of the infection through voluntary vaccination^24^.

### Simulation results in structured populations

**Figure 2:**
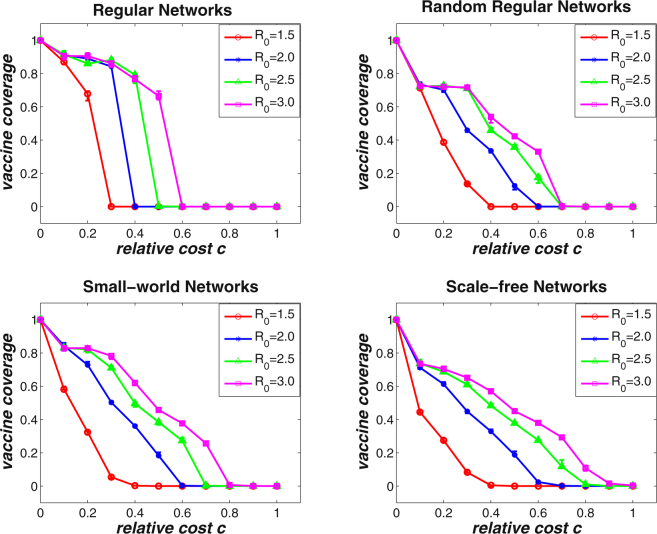
The final vaccine coverage level with respect to different basic reproduction number *R*_0_ for varying relative cost *c*. Simulations are carried out on regular networks (upper left), random regular networks (upper right), small-world networks (bottom left), and scale-free networks (bottom right). The results are averaged over 50 independent network simulations with network size *N* = 5000 and average degree 〈*k*〉 = 4. It can be observed that given a relative cost *c*, the larger the basic reproduction number *R*_0_ is, the higher the final vaccine coverage level can be reached.

The basic reproduction number *R*_0_ represents the average number of secondary cases caused by one primary infection over the courses of its infectious period, which can be used to indicate the severity of an epidemic. Along this line, we aim to evaluate the effects of disease severity on the final voluntary vaccine coverage in different types of structured populations. Figure 2 demonstrates the simulation results of the proposed evolutionary N-person threshold game on four types of complex networks with respect to different disease severity levels, which are measured by *R*_0_ = 1.5, *R*_0_ = 2.0, *R*_0_ = 2.5, and *R*_0_ = 3.0. The results are averaged over 50 independent network simulations with network size *N* = 5000 and average degree 〈*k*〉 = 4. It can be observed that irrespective of the network structure and the disease severity, the final vaccine coverage level gradually decreases as the relative cost *c* becomes larger. Moreover, similar to the situations in well-mixed populations, the vaccine coverage level cannot reach 100% only when the relative cost *c* is zero (i.e., the cost of vaccination is negligible). Another observation is that when the relative cost *c* is large enough, the vaccine coverage level drops to zero. However, when that the basic reproduction number *R*_0_ is larger (i.e., the disease severity is high), the vaccine coverage decreases more lowly with the increase of relative cost *c*. It is reasonable that the more serious the disease, the greater the infection risk individuals will face. In other words, as *R*_0_ increases, the expected payoff for unvaccinated individuals decreases, which makes individuals prone to vaccinate. It can also be observed that as the network structure becomes more diverse (e.g., from regular to scale-free networks), the downward trend of the final vaccine coverage level becomes moderate when the relative cost *c* increases. The major reason is that for diverse networks (e.g., scale-free networks), the group size of each game varies greatly, which makes vaccinating decisions with high payoff propagate more easily by means of the birth-death process.

**Figure 3:**
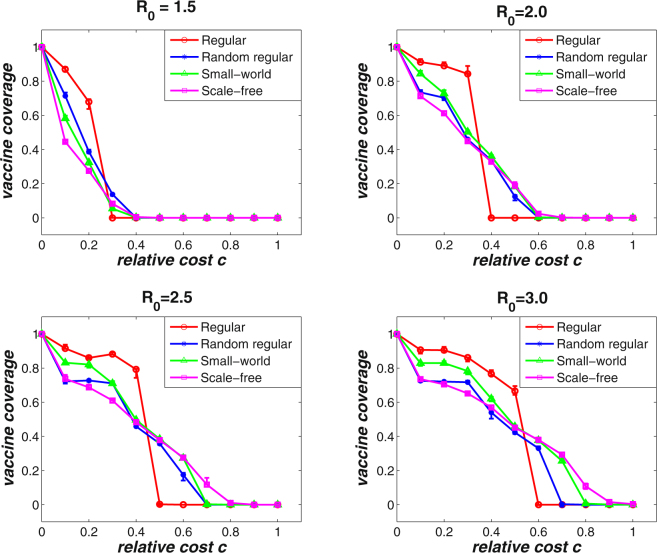
The effects of population structure on the final vaccine coverage level for varying relative cost *c*. The simulations are conducted for *R*_0_ = 1.5 (upper left), *R*_0_ = 2.0 (upper right), *R*_0_ = 2.5 (bottom left), and *R*_0_ = 3.0 (bottom right), respectively. The results are averaged over 50 independent network simulations with network size *N* = 5000 and average degree 〈*k*〉 = 4. As the relative cost *c* increases from zero to a critical value, the final vaccine coverage drops from 100 percent to 0 for all types of networks. It can be observed that the more diverse the network becomes (e.g., scale-free networks), the larger the critical relative cost will be.

Evidence has shown that structural diversity of complex networks can promote the emergence of cooperative behaviors in the face of public dilemma. To identify the effects of population structure (i.e., network structure) on the voluntary vaccinating behaviors in the proposed N-person threshold game, we carry out simulations on four types of complex networks, they are, regular networks, random regular networks, small-world networks, and scale-free networks. Figure 3 shows the effects of population structure on the final vaccine coverage level with respect to different values of basic reproduction number *R*_0_. As before, the results are averaged over 50 independent network simulations with size *N* = 5000 and average degree 〈*k*〉 = 4. Unsurprisingly, for each kind of networks, the final vaccine coverage level will drop to zero when the relative cost *c* is larger than a critical value *c*_*T*_. For example, when *R*_0_ = 3.0 (the bottom right subfigure in Fig. 3), the vaccine coverage level will reach zero on small-world networks when the relative cost is greater than *c*_*T*_ = 0.8. Moreover, as the population structure becomes diverse, the value of *c*_*T*_ will increases. For example, when *R*_0_ = 3.0, *c*_*T*_ increases from 0.6, 0.7, 0.8 to 1.0 for regular, random regular, small-world, and scale-free networks, respectively. More interestingly, it can also be found that the red curves (i.e., results on regular networks) intersects with all other curves before it reaches to zero. In other words, when the relative cost is small, networks with low structural diversity (e.g., regular networks) can more easily maintain a high level vaccine coverage than networks with high structural diversity (e.g., scale-free networks). However, as the relative cost increases, the vaccine coverage level decreases more abruptly for networks with low structural diversity. It means that the diversity of group size may slow down the downward trend of vaccine coverage as the relative cost increases.

**Figure 4:**
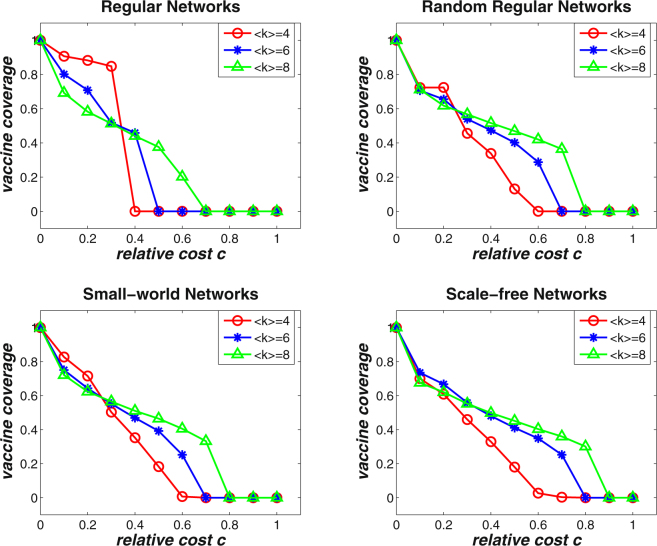
The effects of average degree on the final vaccine coverage level for varying relative cost *c* on regular networks, random regular networks, small-world networks, and scale-free networks. The basic reproduction number is *R*_0_ = 2.0. The results are averaged over 50 independent network simulations with size *N* = 5000. When the relative cost is small, the final vaccine coverage level is lower for networks with larger average degree than those with smaller average degree; as the relative cost increases, the vaccine coverage level deceases more quickly for networks with smaller average degree.

In a structured population, the more connectivities an individual has, the more groups s/he belongs to. According to the simulation procedure in this paper, individuals with higher degree in a network will simultaneously participate into more threshold games. To investigate the effects of average degree on the evolution of vaccinating behaviors, we conduct simulations on four types of complex networks with average degree 〈*k*〉 = 4, 〈*k*〉 = 6, and 〈*k*〉 = 8. Figure 4 shows the simulation results of the final vaccine coverage level with respect to varying relative cost, which are averaged over 50 independent network simulations with size *N* = 5000. It can be observed that when the relative cost *c* is small, the final vaccine coverage level is lower for networks with larger average degree than those with smaller average degree. While as the relative cost increases, the vaccine coverage level deceases more quickly for networks with smaller average degree. Networks with higher average degree can maintain nonzero vaccine coverage for even larger relative cost (i.e., the critical value *c*_*T*_). Moreover, the increasing average degree can also stabilize the downward trend of vaccine coverage level as the relative cost increases. The reason may be that in networks with high average degree, each individual may participate in more threshold games than those in networks with low average degree. In other words, the networks with higher average degree are more diverse in terms of the number of groups an individual may belongs to. Therefore, both the diversity in group size and in the number of games in which each individual participates can affect the voluntary vaccinating behaviors in structured populations.

**Figure 5:**
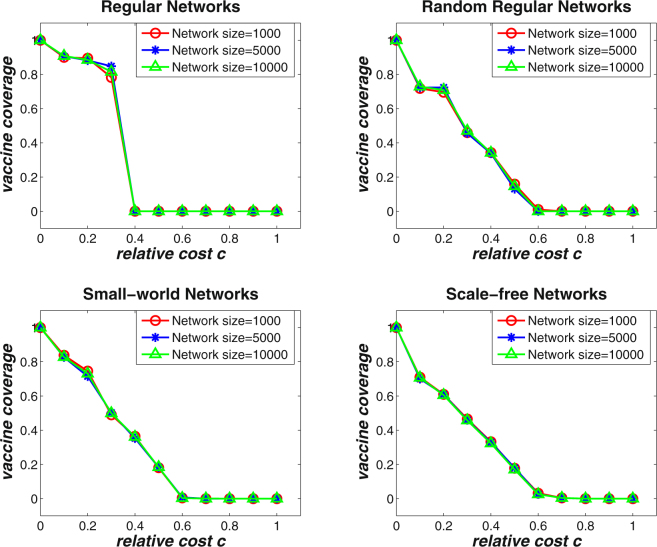
The effects of network size on the final vaccine coverage level for varying relative cost *c* on regular networks, random regular networks, small-world networks, and scale-free networks with average degree 〈*k*〉 = 4. The basic reproduction number is *R*_0_ = 2.0. The network size is set to be *N* = 1000, *N* = 5000, and *N* = 10000, respectively. It can be observed that the final vaccine coverage level are consistent for all types of networks with different size.

**Figure 6:**
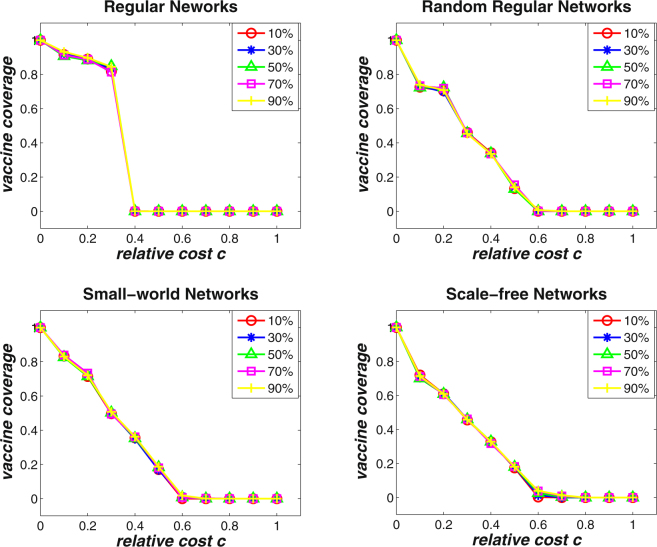
The effects of initially vaccinated individuals on the final vaccine coverage level for varying relative cost *c* on regular networks, random regular networks, small-world networks, and scale-free networks with average degree 〈*k*〉 = 4. The percentage of initially vaccinated individuals is set to be 10%, 30%, 50%, 70%, and 90%, respectively. The basic reproduction number is *R*_0_ = 2.0. It can be observed that the results are consistent for different percentages of initially vaccinated individuals on the four types of networks.

In well-mixed populations, analytical results have shown that population size has remarkable impacts on the stable equilibrium of vaccine coverage level under different relative cost *c* (see Fig. 1). Along this line, it would be necessary to investigate the evolutionary dynamics of voluntary vaccinating behaviors on various networks with different sizes. Figure 5 shows the effects of network size on the final vaccine coverage level on four types of complex networks. The network size is set to be *N* = 1000, *N* = 5000, and *N* = 10000, respectively, and the average degree of the networks is set to be 〈*k*〉 = 4. It can be found that the final vaccine coverage levels are consistent for all types of networks with different sizes. In conjunction with the observations in Fig. 4, it can be deduced that it is the diversity on individual connectivity rather than the size of the overall network that has an effect on the final vaccination outcome. Further, simulations are also carried out to evaluate the effects of the initial fraction of vaccinated individuals on the final outcomes. Figure 6 demonstrates the simulation results on the four types of networks with average degree 〈*k*〉 = 4, where the percentage of initially vaccinated individuals is set to be 10%, 30%, 50%, 70%, and 90%, respectively. It can be observed that similar results can be achieved under different settings of initially vaccinated individuals irrespective of the network structure. Such an observation indicates that given the severity of an epidemic, the evolutionary dynamics of voluntary vaccinating behaviors at the population level is determined mainly by population structure no matter individuals’ initial willingness to vaccinate.

## Discussion

In recent years, many studies have focused on investigating whether or not the critical vaccine coverage necessary for disease elimination can be reach through individuals’ voluntary vaccination. Accordingly, various approaches have been proposed to model the interaction between the dynamics of disease epidemic and the behavioral responses of human beings, among which the game-theoretic approach is widely adopted to analyze individuals’ rational behaviors according to self-interest^17–23^. In most vaccination games, individuals are assumed to be rational and make vaccinating decisions through weighing the cost of vaccination and the risk of infection against the disease prevalence. Along this line, in this paper we model individuals’ vaccinating behaviors by integrating the calculation of infection risk and the *HIT* into the payoff matrix of an evolutionary N-person threshold game. Specifically, the infection risk of an individual depends on the vaccine coverage level in a well-mixed population, or in his/her neighborhood in a structured population. While the *HIT* is determined by both the population/neighborhood size and disease severity. In doing so, individuals’ vaccinating behaviors can dynamically evolve to respond to the disease epidemic based on their strategic updating rules.

Previous game-theoretic models using voluntary vaccination programs have shown that there is a clash between individuals’ self-interest and the group interest in a finite well-mixed populations^17,18^. Their results indicate that it would not be possible to eradicate a vaccine-preventable disease unless a risk-free vaccine is used. In their analysis, individuals adopting the mixed vaccinating strategy, can fully interact with each other without limitation in a well-mixed population. While in this paper, individuals adopting the the pure vaccinating strategy (i.e., whether or not to vaccinate), are assumed to know the herd immunity threshold. Moreover, a structured population is assumed where each time only a small number of individuals can fully interact with each other. Such assumptions are more realistic because people often contact with each other in groups, such as families and colleagues. In doing so, we have revealed the relationships among the population size, the basic reproduction number, and the vaccine coverage level at equilibrium. We have further shown that the full vaccine coverage can be achieved in a population when the cost of vaccination is free. However, as the relative cost of vaccination and infection increases, the vaccine coverage at equilibrium may decrease rapidly. Moreover, the larger the well-mixed interaction group is (i.e., as *N* increases in Fig. 1), the higher level of vaccine coverage can be reached for the population as a whole.

In a certain sense, to explore individuals’ vaccinating behaviors under voluntary vaccination is very similar to study the emergence and evolution of human cooperative behaviors in the face of a public dilemma. For many years, special attentions have been paid to investigate the evolution of cooperation in two-person games, such as the prisoner’s dilemma^55,56^, the stag-hunt game^57^, and the snowdrift game^58,59^. Researchers have found that population structure can remarkably influence the evolution of cooperation under standard two-person games^60–64^. However, the solution for many real-world problems depends on collective behaviors of multiple participants. In this case, some standard two-person games have been generalized to involve multiple participants^65–68^. For example, Zheng *et al*. have extended the snowdrift game to be an evolutionary N-person snowdrift game^65^. Su *et al*. have studied the effects of spatial structure on the evolution of cooperation under N-person snowdrift games^68^. Further, with respect to public goods games (PGGs), many studies have also shown that social diversity (e.g., population structure) can promote the emergence of cooperation^51,69,70^. Although the game in this paper is different, we have similar observations that both diversity in population structure and the average group size of each game have remarkable effects on the final vaccine coverage level. Moreover, we have also found that such effects depend largely on the relative cost of vaccination and infection.

The framework of N-person threshold games aims to characterize the real-world public dilemma that no common benefit can be produced unless its cost is shared by a minimum number of cooperating individuals^49,50,71–73^. For example, in the generalized N-person snowdrift game, if individuals do not have the ability to clear the snow alone, at least two individuals are required to cooperate with each other to shovel the snow. Accordingly, the cost of producing the common benefit is shared by all cooperators. Along this line, the evolution of cooperation under N-person snowdrift games with threshold have been studied in both well-mixed populations^49^ and structured populations^50^. Further, Mikkelsen and Bach have argued that due to the existence of thresholds, the game cannot be represented as the sum of pairwise interactions among participants^73^. Different from existing studies, in this paper, each cooperative (i.e., vaccinated) individual must afford a fixed cost (i.e., the cost of vaccination *c*_*v*_) rather than sharing the cost with other cooperators. All individuals will benefit from herd immunity only when the vaccine coverage level exceeds a threshold value, and their payoffs depend nonlinearly on the number of cooperators in their neighboring environments. In another sense, the proposed N-person threshold game offers a new perspective to investigate the evolution of cooperation in the face of social dilemma of voluntary vaccination.

The main purpose of our analysis and simulations is to explore voluntary vaccinating behaviors from the perspective of evolutionary game theory. We have presented results under different parameter settings in both well-mixed and structured populations. However, there still have several limitations in this work. First, the risk of infection *r*_*p*_ of the unvaccinated individuals is deduced based on the simple SIR model. Although the same result can also be derived from the SIR model with a constant birth/death rate^18^ and the SEIR model^74^, more complicated disease transmission models are needed to characterize the complex interplay between diseases epidemics and human behavioral response. Second, it is assumed that all individuals are rational to make vaccinating decisions. However, in reality, people often tend to exaggerate the negative effects of vaccination failure and complications. Therefore, it would be essential to study individuals vaccinating behaviors with bounded rationality^75^, as well as the effects of their memory and adaptability for past vaccinating events^22^. Third, it is also assumed that all individuals know the severity of the disease (i.e., the basic reproduction number *R*_0_). However, in reality, individuals may not know exactly the disease severity. Usually, they perceive the risk of infection through interacting with their social neighbors^33^, or based on their awareness about the disease prevalence^22^. Fourth, in this paper, individuals adjust their vaccinating by means of imitating one of his/her social neighbors. In the future, several evolutionary strategies can be systematically investigated in the proposed framework, such as imitation^52–54^, pairwise comparison^74^, birth-death and death-birth strategies^76^. Last but not least, we do not consider any incentive-based vaccination programs in this work. Built upon the proposed game-theoretic framework, several types of incentive mechanisms^21,22,53^, can be involved to investigate how the use of incentives influences human vaccinating behaviors, which is worthy of being pursued in the future.

## Methods

### SIR model and herd immunity threshold

Epidemiological evidence shows that individuals who are immune to a disease can slow or prevent the transmission of the disease to others^77^. Accordingly, there exists a public-goods dilemma: the greater the proportion of vaccinated individuals in a population, the less likely those who are not vaccinated are to be infected. In this paper, the classical Susceptible-Infected-Recovered (SIR) model is adopted to simulate the transmission dynamics of infectious diseases^17–20,78^. In the SIR model, the fraction of susceptible (*S*), infected (*I*), and recovered (*R*) individuals dynamically evolves based on the following deterministic ordinary differential equation:

1 $$\frac{dS}{dt}=-\beta SI,$$

2 $$\frac{dI}{dt}=\beta SI-\gamma I,$$

3 $$\frac{dR}{dt}=\gamma I,$$

where *β* is the number of effective contacts per susceptible individual per day that are sufficient to spread the disease, and *γ* is the recovery rate. When the proportion of vaccinated individuals reaches a critical value *p*_*c*_, called the herd immunity threshold (*HIT*), the disease may no longer persist in the population^13,15^. Mathematically, we have $\beta \mathrm{(1}-{p}_{c})/\gamma =1$, that is, ${p}_{c}=1-\mathrm{1/}{R}_{0}$. Here, the basic reproduction number *R*_0_ = *β*/*γ*, which represents the number of cases one primary infection caused on average over the course of its infectious period in an entirely susceptible and well-mixed population^15,24^.

### Voluntary vaccination and N-person threshold games

With respect to voluntary vaccination, individuals usually make vaccinating decisions through evaluating the relative cost of vaccination and infection. Due to the effects of herd immunity, unvaccinated individuals (i.e., free-riders) will benefit from other individuals’ vaccinating behaviors without affording the cost of vaccination. In this case, the value of *HIT p*_*c*_ can be treated as a threshold under which all unvaccinated individuals have risk of being infected. We use *c*_*v*_ and *c*_*i*_ to denote the cost of vaccination and infection, respectively^18^. In doing so, given a population of size *N*, if the number of vaccinated individuals *k* is greater than the critical value *N* · *p*_*c*_, the payoff of a vaccinated individual is −*c*_*v*_, and that of an unvaccinated individual is 0. Otherwise, the payoff of a vaccinated individual is still −*c*_*v*_, but the payoff of an unvaccinated individual is $-{r}_{p}{c}_{i}$. Here, *r*_*p*_ represents the probability that an unvaccinated individual will eventually be infected when the proportion of vaccinated individuals in the population is *p* = *n*_*v*_/*N*. In Table 1, we summarize the payoffs of vaccinated and unvaccinated individuals in both cases.

**Table 1:** Payoff values for the N-person threshold game with herd immunity threshold *p*_*c*_.

Payoff obtained	Vaccinated	Unvaccinated
0 < *n*_*v*_ < *Np*_*c*_	−*c*_*v*_	−*r*_*k*_ *c*_*i*_
*n*_*v*_ ≥ *Np*_*c*_	−*c*_*v*_	0

In a well-mixed population, given the vaccine coverage level *p*, the infection risk *r*_*p*_ of unvaccinated individuals can be calculated based on various epidemic models^18,22,52^. For the SIR model in this paper, we have previously deduced that *r*_*p*_ satisfies^33^:

4 $${r}_{k}=1-\frac{1}{{R}_{0}\mathrm{(1}-p)}=1-\frac{N}{{R}_{0}(N-{n}_{v})}.$$

Notice that the same result has been derived by Bauch and Earn for the SIR model with constant birth and death rate^18^, and similar results have also been proposed for the SEIR model^79^. In doing so, an individual’s voluntary vaccinating decision depends not only on the relative cost of vaccination and infection *c* = *c*_*v*_/*c*_*i*_, but also the decisions of other individuals in the population (i.e., the vaccination coverage *p*).

### Equilibrium analysis in well-mixed populations

Suppose a group of *N* individuals are sampled from a very large population (*Z *→ ∞), where a fraction *x* is composed of vaccinated individuals (*V*), the remaining (1−*x*) being the fraction of unvaccinated individuals (*U*). Then, the fitness of vaccinated and unvaccinated individuals can be determined based on a binomial sampling^49,65,80^, that is,

5 $${f}_{V}(x)=\sum _{{n}_{v}\mathrm{=0}}^{N-1}(\begin{array}{c}N-1\\ {n}_{v}\end{array}){x}^{{n}_{v}}{\mathrm{(1}-x)}^{N-1-{n}_{v}}{P}_{V}({n}_{v}+\mathrm{1)}={P}_{V}({n}_{v}+\mathrm{1),}$$

and

6 $${f}_{U}(x)=\sum _{{n}_{v}\mathrm{=0}}^{N-1}(\begin{array}{c}N-1\\ {n}_{v}\end{array}){x}^{{n}_{v}}{\mathrm{(1}-x)}^{N-1-{n}_{v}}{P}_{U}({n}_{v}),$$

where ${P}_{V}({n}_{v}+\mathrm{1)}=-{c}_{v}$ is the payoff of vaccinated individuals when their number is ${n}_{v}+1$, ${P}_{U}({n}_{v})=$ $(-{r}_{k}{c}_{i})\cdot \delta (N{p}_{c}-{n}_{v})$is the payoff of unvaccinated individuals when the number of vaccinated individuals is *k*. Here, *δ*(*x*) is a Heaviside step function satisfies *δ*(*x* > 0) = 1 and *δ*(*x* ≤ 0) = 0. Based on the replicator equation^49,65^, previous studies have shown that there exists an interior stable state of *x* ^*^, satisfying ${f}_{V}({x}^{\ast })-{f}_{U}({x}^{\ast })=0$. Combing Equations (5) with (6), we have

7 $${f}_{V}(x)-{f}_{U}(x)=\sum _{{n}_{v}\mathrm{=0}}^{M-1}(\begin{array}{c}N-1\\ {n}_{v}\end{array}){x}^{{n}_{v}}{\mathrm{(1}-x)}^{N-1-{n}_{v}}{r}_{k}{c}_{i}-{c}_{v}=\mathrm{0,}$$

where *M* = *Np*_*c*_ represents the minimum number of vaccinated individuals required to reach the *HIT*. Therefore, the stable fixed point *x* ^*^ satisfies the following equation

8 $$\sum _{{n}_{v}\mathrm{=0}}^{M-1}(\begin{array}{c}N-1\\ {n}_{v}\end{array}){x}^{\ast {n}_{v}}{\mathrm{(1}-{x}^{\ast })}^{N-1-{n}_{v}}{r}_{k}{c}_{i}-{c}_{v}=0.$$

That is

9 $$\sum _{{n}_{v}\mathrm{=0}}^{M-1}(\begin{array}{c}N-1\\ {n}_{v}\end{array}){x}^{\ast {n}_{v}}{\mathrm{(1}-{x}^{\ast })}^{N-1-{n}_{v}}{r}_{k}=c,$$

where *c* = *c*_*v*_/*c*_*i*_ is the relative cost of vaccination and infection.

Let us start from a sufficiently small value of *R*_0_ such that *M* = *N *· *p*_*c*_ = 1, which means that only one vaccinated individual is required to reach the *HIT*. In this case, Equation (9) becomes ${\mathrm{(1}-{x}^{\ast })}^{N-1}{r}_{0}=c$, where *r*_0_ = 1−1/*R*_0_ is calculated from Equation (4) when *n*_*v*_ = 0. Thus, when *M* = 1, the stable fixed point *x* ^*^ satisfies the following equation

10 $$(1-\frac{1}{{R}_{0}}){\mathrm{(1}-{x}^{\ast })}^{N-1}=c.$$

When *N* = 2, we have $1-\mathrm{1/}{R}_{0}=\mathrm{1/}N$ based on *Np*_*c*_ = *M*. In this case, we find that ${x}^{\ast }=1-2c$ is a stable fixed point. Similarly, when *M* = 2, we have

11 $${r}_{0}{\mathrm{(1}-{x}^{\ast })}^{N-1}+{r}_{1}(N-\mathrm{1)}{x}^{\ast }{\mathrm{(1}-{x}^{\ast })}^{N-2}=c,$$

where *r*_0_ and *r*_1_ can be calculated from Equation (4), and *R*_0_ can be derived from $N\mathrm{(1}-\mathrm{1/}{R}_{0})=M$. For *N* > 3 and *M* > 2, it is hard to get analytical solutions. However, we can solve Equation (9) numerically.

### Evolutionary dynamics in structured populations

In reality, infectious diseases spread in the crowd through individual interactions, where people are often structured in groups, such as families, classmates, and colleagues. Many studies have focused on studying evolutionary dynamics of group interactions on top of structured populations^34,35,50,51^. For example, the seminal study by Santos *et al*.^51^ has reformulated the public goods game to be staged on complex networks. Motivated by this consideration, in this paper we simulate the N-person threshold games on four types of complex networks (i.e., regular networks, random regular networks, small-world networks, and scale-free networks), by assuming that each individual together with his/her neighbors forms a local well-mixed population group. Specifically, each individual *i* with degree *k*_*i*_ in a given network can participate in *k*_*i*_ + 1 games in different groups, where one group is centered on himself/herself and the other *k*_*i*_ games are centered on his/her *k*_*i*_ neighbors (see Fig. 1 in reference^51^). In Table 2, we summarize the payoff values of the proposed game within the focal group of individual *i*.

**Table 2:** Payoff values for the N-person threshold game within the focal group of individual *i* with degree *k*_*i*_.

Payoff obtained	Vaccinated	Unvaccinated
0 < *n*_*v*_ < (*k*_*i*_ + 1)*p*_*c*_	−*c*_*v*_	−*r*_*k*_ *c*_*i*_
*n*_*v*_ ≥ (*k*_*i*_ + 1)*p*_*c*_	−*c*_*v*_	0

To explore the evolution of individuals’ vaccinating behaviors, the simulation procedure is conducted as follows. Initially, a faction of individuals is randomly vaccinated in the population. Each individual *i* calculates its average payoff values over *k*_*i*_ + 1 games s/he participates based on the proposed threshold games. Then, individuals’ vaccinating strategies are updated by means of a birth-death process, combined with the pairwise comparison rule^74,81^. At each round of the birth-death process, all individuals will simultaneously update their strategies by comparing their fitness at the current round with the fitness of a randomly chosen neighbor at the previous round. Specifically, an individual *i* imitates the strategy of a randomly selected neighbor *j* with a probability calculated by a Fermi function:

12 $${p}_{i\to j}=\frac{1}{1+\exp [-\eta ({f}_{j}-{f}_{i})]}$$

where *η* represents the strength of selection, and *f*_*i*_ (respectively, *f*_*j*_) is the fitness of individual *i* (respectively, *j*). Throughout the simulations in this paper, *η* is set to be 10, which indicates a strong selection^52^. In this paper, we will evaluate the effects of basic reproduction number *R*_0_, network structure, and average degree on the evolutionary dynamics of voluntary vaccination in terms of different relative costs of vaccination and infection *c*. Moreover, we will also exam whether or not the initial settings including network size can affect the final evolutionary stable states.
